# The Disease Severity and Clinical Outcomes of the SARS-CoV-2 Variants of Concern

**DOI:** 10.3389/fpubh.2021.775224

**Published:** 2021-11-30

**Authors:** Lixin Lin, Ying Liu, Xiujuan Tang, Daihai He

**Affiliations:** ^1^Department of Applied Mathematics, The Hong Kong Polytechnic University, Kowloon, Hong Kong SAR, China; ^2^School of International Business, Xiamen University Tan Kah Kee College, Zhangzhou, China; ^3^Shenzhen Center for Disease Control and Prevention, Shenzhen, China

**Keywords:** SARS-CoV-2, variants of concerns, disease severity, mortality, epidemic potential, COVID-19

## Abstract

With the continuation of the pandemic, many severe acute respiratory syndrome coronavirus 2 (SARS-CoV-2) variants have appeared around the world. Owing to a possible risk of increasing the transmissibility of the virus, severity of the infected individuals, and the ability to escape the antibody produced by the vaccines, the four SARS-CoV-2 variants of Alpha (B.1.1.7), Beta (B.1.351), Gamma (P.1), and Delta (B.1.617.2) have attracted the most widespread attention. At present, there is a unified conclusion that these four variants have increased the transmissibility of SARS-CoV-2, but the severity of the disease caused by them has not yet been determined. Studies from June 1, 2020 to October 15, 2021 were considered, and a meta-analysis was carried out to process the data. Alpha, Beta, Gamma, and Delta variants are all more serious than the wild-type virus in terms of hospitalization, ICU admission, and mortality, and the Beta and Delta variants have a higher risk than the Alpha and Gamma variants. Notably, the random effects of Beta variant to the wild-type virus with respect to hospitalization rate, severe illness rate, and mortality rate are 2.16 (95% CI: 1.19–3.14), 2.23 (95% CI: 1.31–3.15), and 1.50 (95% CI: 1.26–1.74), respectively, and the random effects of Delta variant to the wild-type virus are 2.08 (95% CI: 1.77–2.39), 3.35 (95% CI: 2.5–4.2), and 2.33 (95% CI: 1.45–3.21), respectively. Although, the emergence of vaccines may reduce the threat posed by SARS-CoV-2 variants, these are still very important, especially the Beta and Delta variants.

## Introduction

There are multiple severe acute respiratory syndrome coronavirus 2 (SARS-CoV-2) variants identified by viral genomic sequencing in different parts of the world. Based on the potential threats of these viral variants in terms of transmission, disease severity, immune escape, etc., they were classified into variants of concern (VOCs) and variants of interest (VOIs) by the World Health Organization (WHO). So far, four variants have been defined as VOCs—Alpha (B.1.1.7), Beta (B.1.351), Gamma (P.1), and Delta (B.1.617.2).

In late December 2020, the Alpha variant was reported in the United Kingdom (1), followed quickly by the detection of the Beta variant, which carried three mutations including K417N, E484K, and N501Y at important locations in the Spike protein receptor-binding domain (S-RBD) in South Africa (2). In early January 2021, the Gamma variant carrying three mutations consist of K417T, E484K, and N501Y in the S-RBD was reported in Brazil (3). In December 2020, the Delta variant carrying mutations called 452R and 478K was first isolated in India (4).

Three key concerns of SARS-CoV-2 VOCs are viral transmissibility, disease severity, and the impacts on vaccine efficacy. For viral transmissibility, the reported studies have yielded good evidence that all VOCs are more transmissible than the wild-type virus (5–13). Risk of transmission, reported in 15 studies, was 45–71% higher for Alpha variant than the wild-type virus, while the basic reproduction number *R*_0*t*_ was 75–78% higher than the wild-type virus, and the reported effective reproduction number *R*_*t*_ ranged from 1.1 to 2.8 (8). For Beta variant, the *R*_*t*_ was 1.55 (95% confidence interval [CI]: 1.43–1.69) and ~50% more transmissible than the previously circulating variants (11, 12). Using dynamic modeling that integrates genomic and mortality data, Faria et al. (13) estimated that the transmissibility of the Gamma variant could be 1.4–2.2 times higher than that of the wild-type virus. A statistically significant increase in *R*_*t*_ relative to wild-type virus of Delta variant at 97% (95% CI: 76–117) (9). For impacts on vaccine effectiveness, the effects of the viral variants on the vaccine's protection of infection, symptomatic disease, and severe disease have been considered. The Alpha variant had less impact on the vaccine, and the vaccine was therefore still protective (14, 15). For Beta variant, the protection offered by the vaccine against symptomatic disease was reduced (16–18). The conclusion on the impact of Gamma variant on the vaccine was not yet clear. Delta variant likely reduced the protective effect of the vaccine with respect to infection and symptomatic disease (19).

Based on the newest report from WHO, the conclusions on disease severity were most uncertain among the reviews focusing on the phenotypic effects of SARS-CoV-2 VOCs. There were few reports on the disease severity of the variant viruses. Clinical outcomes were influenced by factors such as the use of health-care resource, demographic changes, and trends in social behavior (20). To date, we have found few reports of disease severity analysis based on clinical outcomes of the VOCs. By comparing four studies with datasets on the disease severity of infected persons, it was concluded that Alpha variant may not increase the risk of disease severity (21). A meta-analysis of these four studies indicated significantly increased hazard of mortality among patients with COVID-19 infected with Alpha variant relative to those infected with the wild-type virus (22). Alpha, Beta, and Gamma variants had a 1.7-, 3.6-, and 2.6-fold increased risk of hospitalization, and a 2.3-, 3.3-, and 2.2-fold increased risk of admission to the ICU, respectively (23). However, further confirmation in larger studies of Alpha variant as well as other viral variants are needed.

## Methods

This study is a systematic review of current evidence conducted in June 2021 to determine the effects of SARS-CoV-2 VOCs on disease severity and clinical outcomes. The study was guided by the Preferred Reporting Items for Systematic Review and Meta-Analysis Protocols (PRISMA-P) to ensure reliability and validity of the reported results (24).

### Sources of Data

A systematic search was conducted by using search terms in online databases such as PubMed, Medline, and Embase to retrieve all relevant English papers and reports published between June 1, 2020 and October 15, 2021. The search strategy adopted a combination of the following search terms: (B.1.1.7) OR (B.1.351) OR (P.1) OR (B.1.617.2) OR (SARS-CoV-2 Variants of Concern) OR (SARS-CoV-2 VOCs). Related references were also searched in Google Scholar.

### Selection of Research

**Figure 1:**
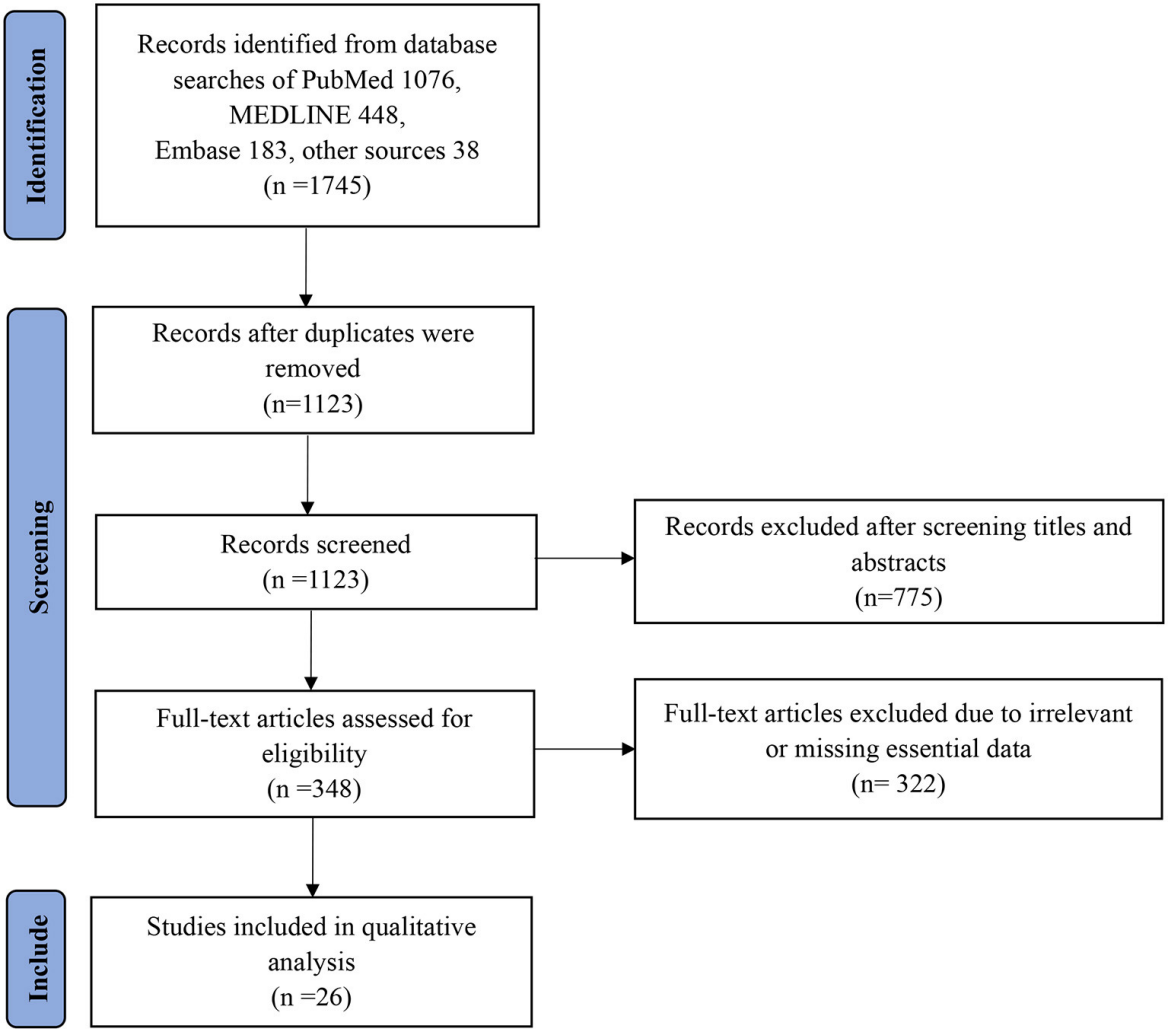
Flow diagram of the search strategy and article selection process.

In all, 1,745 papers were extracted, and the full-text of the most relevant papers based on eligibility criteria were reviewed. Original and peer-reviewed papers in English that met the eligibility criteria in the final report were included. A flow chart of the search strategy and study selection process using PRISMA guidelines is presented in Figure 1. In addition, the following exclusion criteria were used:

- Non-human studies, including animal experiments, *in vitro* observations, and papers that do not refer to the keywords in this review.
- Papers that do not contain data on at least two kinds of viruses.
- The full paper is not available.
- Any duplicate and suspicious results in the database.

### Extraction of Data

The first author's name, variant type, patient recruitment type, study dates, number of participants, age, rate of severe disease, and mortality were recorded in an information sheet. We rechecked the collected data to avoid duplication or overlap. Then, we extracted the relevant data (Table 1).

**Table 1:** Comparison of studies assessing the effect of SARS-CoV-2 VOC on disease severity and clinical outcomes.

**The first author (reference)**	**Variant type**	**Patient recruitment**	**Study dates**	**Number of participants**	**Age of participants, years**	**Primary outcome**	**Rate of Severe disease**	**Rate of mortality**	**Effect on severe disease**	**Effect on mortality**
Frampton et al. (25)	Alpha	Hospital patients with confirmed COVID-19	November 9 to December 20, 2020	341 (69%) included of 496 available patients screened	Median 60 (IQR: 47–75)	Clinical severity as defined by WHO ordinal scale ≥6; mortality at 28 days	0.369	0.162	HR: 1.02 (95% CI: 0.76–1.38)	HR: 1.12 (95% CI 0.71–1.78)
Challen et al. (26)	Alpha	Public health data from community-based testing dataset	Oct 1, 2020, to Jan 28, 2021	109,812 (11.6%) included of 941,518 available patients screened	Mean 46.3 (SD 11.0)	Mortality at 28 days	NA	0.003	NA	HR: 1.64 (95% CI: 1.32–2.04)
Davies et al. (27)	Alpha	Public health data from community-based testing dataset	November 1, 2020 to January 23, 2021	1,146,534 (51.1%) included of 2,245,263 available patients screened	1–34 (44.8%); 35–54 (35.2%); 55–69 (15.3%); 70–84 (3.8%); ≥85 (0.8%)	Mortality at 28 days	NA	0.009	NA	HR: 1.55 (95% CI: 1.39–1.72)
Grint et al. (28)	Alpha	Public health data from community and hospital-based testing dataset	November 16, 2020 to January 11, 2021	184,786 (41.9%) included of 441,161 available patients screened	Median 38.0 (IQR: 24.0–52.0); mean 38.2 (SD: 18.1)	Mortality at 28 days	NA	0.005	NA	HR: 1.67 (95% CI: 1.34–2.09)
Patone et al. (29)	Alpha	Public health data from community-based testing dataset	November 1, 2020 to 26 January, 2021	80,494 (40.6%) included of 198,420 available patients screened	NA	Clinical severity reported by being admitted to CCU: Mortality at 28 days	NA	0.008	HR: 1.99 (95% CI: 1.59, 2.49)	HR: 1.59 (1.25–2.03)
Loconsole et al. (30)	Alpha	Public health data from community-based testing dataset	December 2020 to March 2021	621 (20.2%) included of 3,075 available patients screened	0–4 (3.38%); 5–16 (12.08%);17–35 (23.19%);36–65 (43.8%); >65 (17.55%)	Clinical severity reported by being admitted to Hospital, as severe; Mortality reported	Hospital: 5.6%; Severe: 6.5%	0.6%	HR Hospital: 2; Severe: 1.27	HR: 0.67
Funk et al. (31)	Alpha and Beta and Gamma	Public health data from community-based testing dataset	October 2020 to March 2021	23,343 (0.7%) included of 3,200,000 available patients screened	Alpha: Mean 39 (SD: 21); Beta: Mean 43 (SD: 22); Gamma: Mean 46 (SD: 25);	Clinical severity reported by being admitted to Hospital, being admitted to ICU; Mortality reported	Hospital: 11% (Alpha); 19.3% (Beta); 20% (Gamma); ICU: 1.4% (Alpha); 2.3% (Beta); 2.1% (Gamma)	0.02 (Alpha); 0.052 (Beta); 0.039 (Gamma)	HR Hospital (Alpha): 1.7 (95% CI: 1.0–2.9), (Beta): 3.6 (95% CI: 2.1–6.2); (Gamma): 2.6 (95% CI: 1.4–4.8); ICU (Alpha): 2.3 (95% CI: 1.4–3.5); (Beta): 3.3 (95% CI: 1.9–5.7); (Gamma): 2.2 (95% CI: 1.8–2.9)	HR (Alpha): 0.5 (95% CI: 0.3–0.9), (Beta): 1.1 (95% CI: 0.4–3.4), (Gamma): 0.6 (95% CI: 0.3–1.0)
Public Health England (32)	Alpha and Delta	Public health data from community-based testing dataset	March, 2021 to May, 2021	38,805	NA	Clinical severity reported by being admitted to Hospital, being admitted to emergency care attendance or hospitalization	NA	NA	HR Hospital (Delta vs. Alpha): 2.61, (95% CI: 1.56–4.36); care attendance or hospitalization (Delta vs. Alpha): 1.67, (95% CI: 1.25–2.23)	NA
Bager et al. (33)	Alpha	Public health data from community-based testing dataset	January 1 to March 24, 2021	10,544 (20.7%) included of 50,958 available patients screened	0–29 (44.4%) 30–59 (44.3%) ≥60 (11.3%)	Clinical severity reported by being admitted to hospital; Mortality reported	0.054	NA	HR Hospital: 1.42 (95% CI: 1.25–1.60)	NA
Cetin et al. (34)	Alpha	Public health data from community-based testing dataset	April 2020 to March 2021	588 (15.9%) included of 3,707 available patients screened	NA	Clinical severity reported by being admitted to hospital, being admitted to ICU; Mortality reported	Hospital: 0.335, ICU: 0.075	NA	HR Hospital: 2.62; ICU: 1.923	NA
Fisman and Tuite (35)	Alpha and Beta and Gamma and Delta	Public health data from community-based testing dataset	February 7 to June 27, 2021	168,909 (65.47%) included of 257,997 available patients screened	NA	Clinical severity reported by being admitted to hospital, being admitted to ICU; Mortality reported	Hospital (Alpha and Beta and Gamma): 0.054, ICU (Alpha and Beta and Gamma): 0.012; Hospital (Delta): 0.058, ICU (Delta): 0.015	0.009 (Alpha and Beta and Gamma); 0.007 (Delta)	HR Hospital (Alpha and Beta and Gamma): 1.52 (95% CI: 1.42–1.63); (Delta): 2.08 (95% CI: 1.78–2.4); ICU (Alpha and Beta and Gamma): 1.89 (95% CI: 1.67–2.17); (Delta): 3.35 (95% CI: 2.6–4.3)	HR (Alpha and Beta and Gamma): 1.51 (95% CI: 1.3–1.78); (Delta): 2.33 (95% CI: 1.54–3.31)
Freitas et al. (36)	Gamma	Public health data from community-based testing dataset	April 1, 2020 to May 31, 2021 and January 1 to January 31, 2021	6,142 (47.4%) included of 12,958 available patients screened	NA	Clinical severity reported by being admitted to hospital; Mortality reported	Hospital: 0.860	0.597	HR Hospital: 0.914	HR: 1.315
Grint et al. (37)	Alpha	Public health data from community-based testing dataset	November 16, 2020 to April 21, 2021	93,153 (50.29%) included of 185,234 available patients screened	NA	Clinical severity reported by being admitted to hospital; Mortality reported	0.015	0.0027	HR: 1.62 (95% CI: 1.48 −1.78)	HR: 1.73 (95% CI: 1.41–2.13)
Giles et al. (38)	Alpha	Hospitalized patients with confirmed COVID-19	NA	30 (50%) included of 60 available patients screened	NA	Clinical severity as defined by WHO ordinal scale ≥ 6; mortality at 28 day	0.37	0.321	HR: 1.37	HR: 1.551
Hoang et al. (39)	Alpha and Beta and Gamma	Hospitalized patients with confirmed COVID-19	February–May 2020, June–December 2020, January –September 2021	935 (53.16%) included of 1,760 available patients screened	NA	Clinical severity reported by being admitted to hospital, being admitted to ICU; Mortality reported	Hospital (Alpha): 0.249, (Beta): 0.316, (Gamma): 0.2; ICU (Alpha): 0.071, (Beta): 0.092, (Gamma): 0.1	(Alpha): 0.042, (Beta): 0, (Gamma): 0	HR Hospital (Beta vs. Alpha): 1.27, (Gamma vs. Beta): 0.633, (Gamma vs. Alpha): 0.833; ICU (Beta vs. Alpha): 1.314, (Gamma vs. Beta): 1.087, (Gamma vs. Alpha): 1.314	NA
Kim et al. (40)	Alpha	Public health data from community-based testing dataset	September 20 to December 15, 2020	1,769 (50%) included of 3,538 available patients screened	NA	Clinical severity reported by being admitted to hospital; Mortality reported	Hospital: 0.009	0.0089	HR: 0.6	HR: 1.22
Meyer et al. (41)	Alpha	Public health data from community-based testing dataset	January 12 to June 3, 2021	59 (1.66%) included of 3,544 available patients screened	Minimum 0.0 years, maximum 17.8 years	Clinical severity reported by being admitted to hospital, being admitted to ICU	Hospital: 0.153; ICU: 0.017	NA	HR Hospital: 1.89; ICU: NA	NA
Ong et al. (42)	Alpha, Beta, and Delta	The Ministry of Health	January 1 to May 22, 2021	829 (85%) included of 976 available patients screened	NA	Clinical severity reported by being admitted to ICU: Mortality reported	NA	NA	HR (Delta VS wild-type); ICU: 1.88 (95% CI: 0.95–3.76); others No significant difference	HR (Delta vs. wild-type): 1.88 (95% CI: 0.95–3.76); others No significant difference
Martínez-García et al. (43)	Alpha	Hospital patients with confirmed COVID-19	January 2 to April 30, 2021	426 (27.4%) included of 1,555 available patients screened	NA	Clinical severity reported by being admitted to ICU; Mortality reported	19.5%	13.9%	HR ICU: 2.11 (95% CI: 1.55 −2.87)	HR: 0.87 (95% CI: 0.62–1.23)
Yilmaz et al. (44)	Alpha	Public health data from community-based testing Data set	February 2 to February 9, 2021	339 (26.1%) included of 1,300 available patients screened	NA	Clinical severity reported by being admitted to Hospital; in intensive care	Hospital: 3.2%, intensive care: 0.58%	NA	HR Hospital: 47.76%; intensive care: 77.78%	NA
Twohig et al. (45)	Alpha, Delta	The Ministry of Health	January 1 to May 22, 2021	829 (85%) included of 976 available patients screened	NA	Clinical severity reported by being admitted to ICU; Mortality reported	Hospital: 2.3%, emergency care: 3.4%	NA	HR Hospital (Delta vs. Alpha): 2.26 (95% CI: 1.32–3.89); emergency care: 1.7	NA
Veneti et al. (46)	Alpha, Beta	Norwegian Surveillance System for Communicable Diseases	December 28 to May 2, 2021	23,717 (83.8%) included of 28,301 available patients screened	NA	Clinical severity reported by being admitted to hospital, being admitted to ICU	Hospital (Alpha): 3.8%, (Beta): 4.2%, ICU (Alpha): 0.8%, (Beta): 0.9%	NA	HR Hospital (Alpha vs. wild-type): 1.9 (95% CI: 1.6–2.3), (Beta vs. wild-type): 2.4 (95% CI: 1.7–3.3); ICU (Alpha vs. wild-type): 1.8 (95% CI: 1.2–2.8), (Beta vs. wild-type): 2.7 (95% CI: 1.2–6.5)	NA
Patone et al. (47)	Alpha	Public health data from community-based testing Data set	November 1, 2020 to January 27, 2021	117,926 (59.4%) included of 198,420 available patients screened	NA	Clinical severity reported by being admitted to CCU; mortality at 28 day	CCU: 0.4%	0.4%	HR CCU: 2.15 (95% CI: 1.75–2.65)	HR 1.65 (95% CI: 1.36–2.01)
Nyberg et al. (48)	Alpha	Public health data from community-based testing	November 1, 2020 to January 27, 2021	592,409 (70.59%) included of 839,278 available patients screened	NA	Clinical severity reported by being admitted to Hospital; mortality at 28 day	Hospital: 4.7%	0.44%	HR Hospital: 1.52 (95% CI: 1.47–1.57)	HR: 1.59 (95% CI: 1.44–1.74)
Stirrup et al. (49)	Alpha	Hospital patients with confirmed COVID-19	November 16, 2020 to January 10, 2021	1,107 (47.29%) included of 2,341 available patients screened	NA	Clinical severity reported by being admitted to ITU; mortality at 28 day	ITU: 20.35%	19.62%	HR ITU: 1.01 (95% CI: 0.75–1.37)	HR: 1.01 (95% CI: 0.79–1.28)
Whittaker et al. (50)	Alpha	Public health data from community-based testing Data set	December 21, 2020 to April 25, 2021	946 (81%) included of 1,186 available patients screened	NA	Clinical severity reported by being admitted to ICU; Died in hospital	ICU: 18%	6%	HR ICU: 1.125	HR: 1

### Assessment of Quality

This study adhered to the PRISMA guidelines to ensure the quality and accuracy of selected publications and outcomes.

## Results

We identified a total of 1,745 (1,076, PubMed; 448, MEDLINE; 183, Embase; and 38 from other sources) relevant articles, and 1,123 studies were left after removing the duplicates. After excluding 775 articles by title and abstract screening, 348 articles met the conditions for full-text screening. Based on the above exclusion criteria, a further 322 articles were excluded. Ultimately, 26 studies that met the inclusion criteria were selected in this review for further analysis. Each study's main findings are summarized in Table 1 (25–50). Among the 26 studies, most were related to variant Alpha, followed by Beta, Gamma, and Delta.

**Figure 2:**
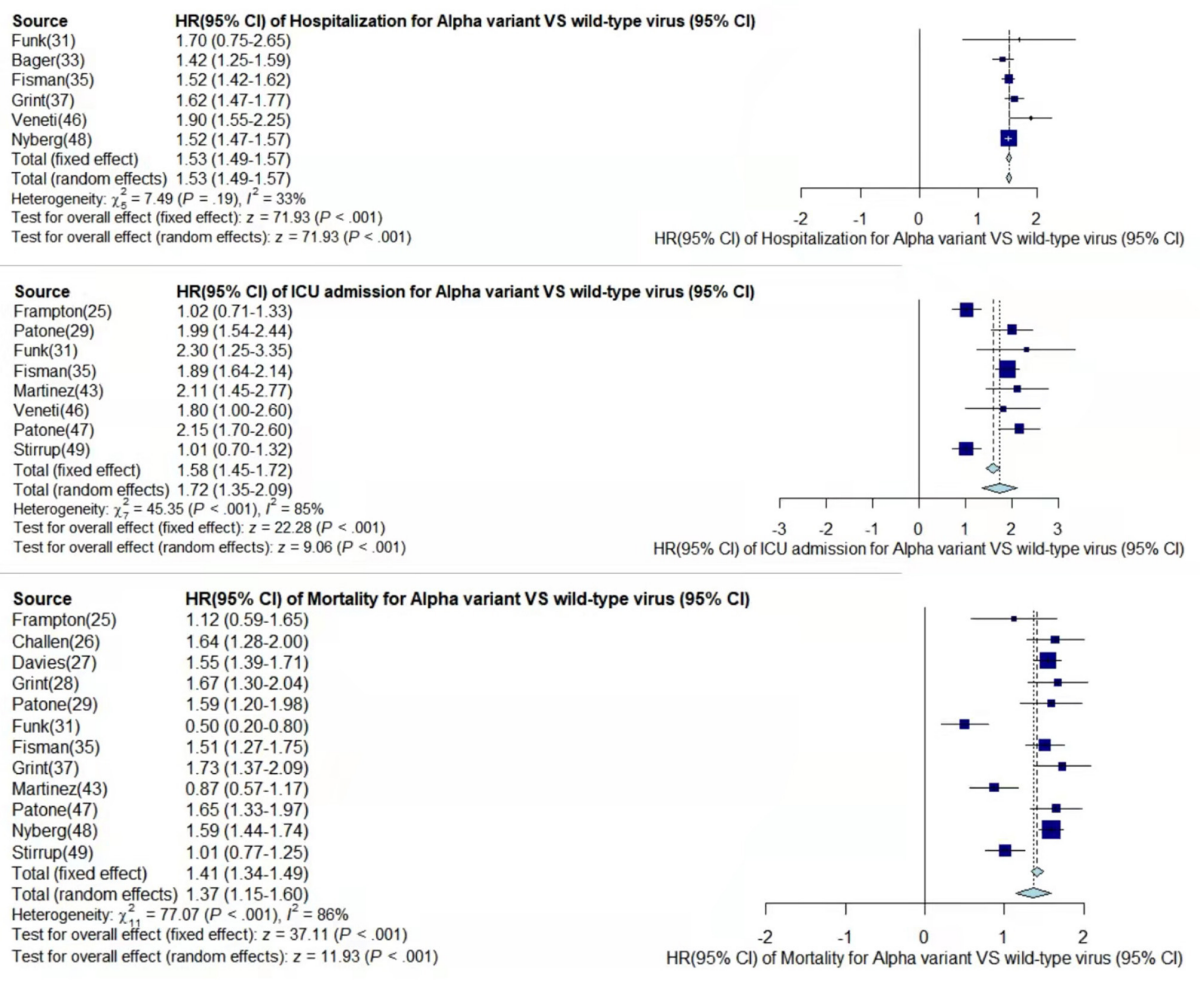
Pooled hazard ratio of hospitalization, ICU admission, and mortality for patients infected with Alpha variant compared to those with wild-type virus.

**Figure 3:**
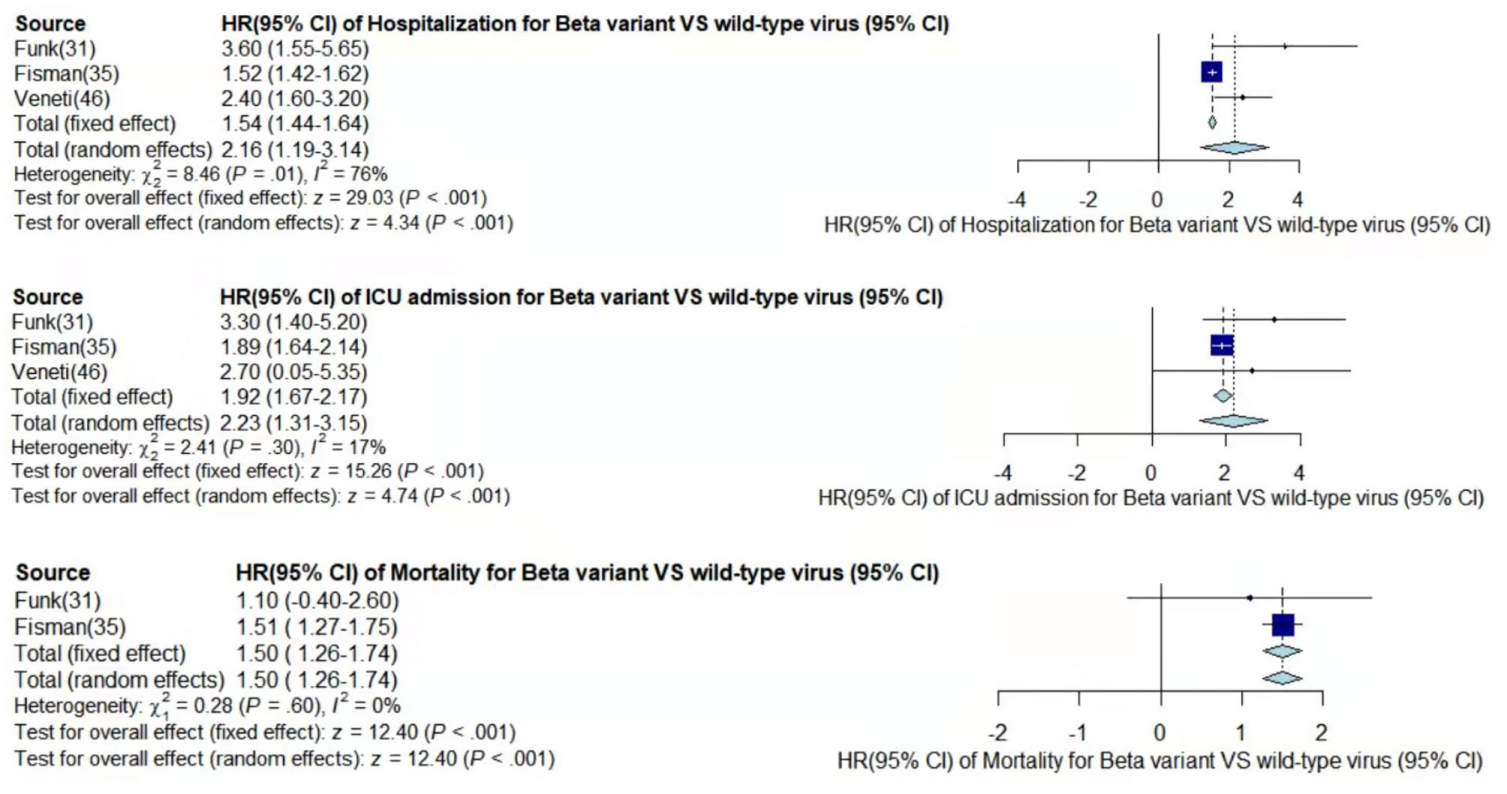
Pooled hazard ratio of hospitalization, ICU admission, and mortality for patients infected with Beta variant compared to those with wild-type virus.

**Figure 4:**
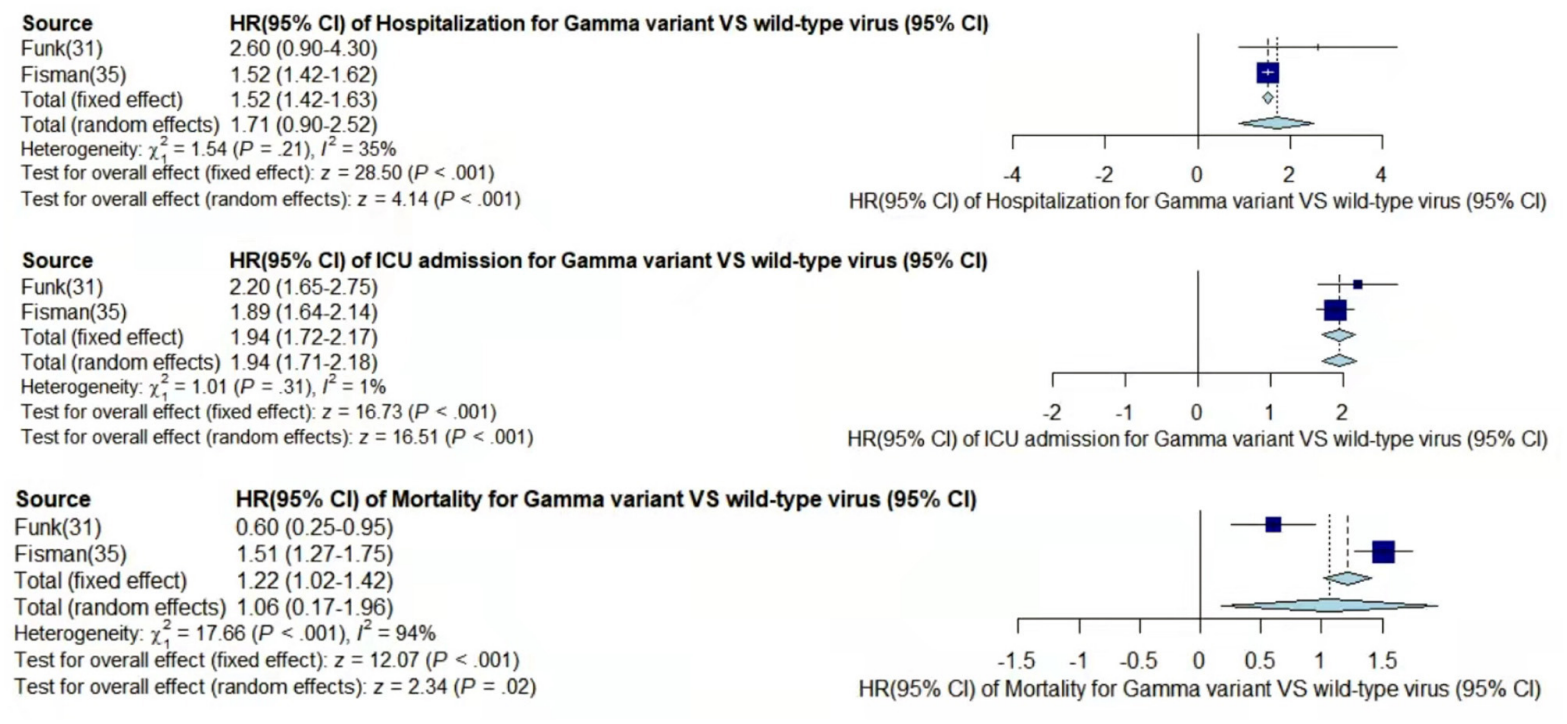
Pooled hazard ratio of hospitalization, ICU admission, and mortality for patients infected with Gamma variant compared to those with wild-type virus.

**Figure 5:**
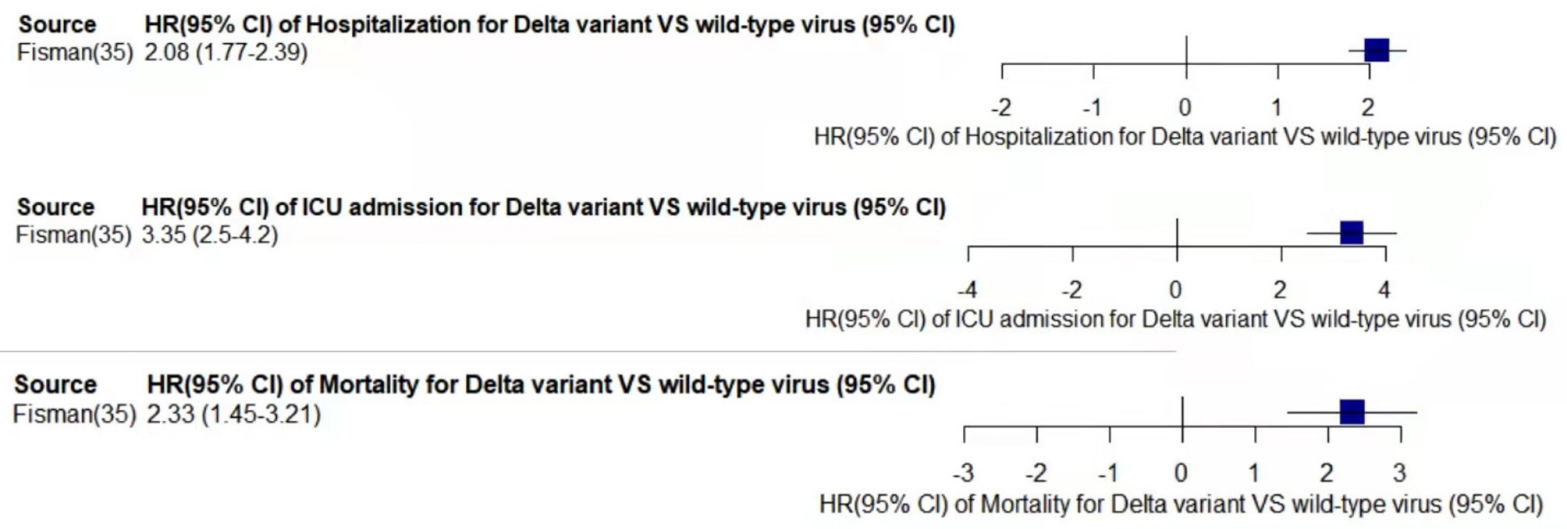
Pooled hazard ratio of hospitalization, ICU admission, and mortality for patients infected with Delta variant compared to those with wild-type virus.

Through meta-analysis, the data provided by the retained studies were integrated; the values of total random effects were retained; and the risk of hospitalization, ICU admission, and mortality of patients infected with VOCs compared with wild-type virus were obtained to analyze the disease severity of the VOCs. The main process of meta-analysis of variants Alpha, Beta, Gamma, and Delta are, respectively, shown in Figures 2–5, and the main results of the meta-analysis are summarized in Table 2.

**Table 2:** Hazard ratios (95% CI) of disease severity of the SARS-CoV-2 VOCs compared with wild-type virus.

	**Alpha**	**Beta**	**Gamma**	**Delta**
Risk of hospitalization	1.53 (95% CI: 1.49–1.57)	2.16 (95% CI: 1.19–3.14)	1.71 (95% CI: 0.9–2.52)	2.08 (95% CI: 1.77–2.39)
Risk of ICU admission	1.74 (95% CI: 1.35–2.09)	2.23 (95% CI: 1.31–3.15)	1.94 (95% CI: 1.71–2.18)	3.35 (95% CI: 2.5–4.2)
Risk of mortality	1.37 (95% CI: 1.15–1.6)	1.50 (95% CI: 1.26–1.74)	1.06 (95% CI: 0.17–1.96)	2.33 (95% CI: 1.45–3.21)

In the case of Alpha variant compared with the wild-type virus, most studies concluded that the risk of hospitalization, ICU admission, and mortality were increased. Only Frampton et al. (25) and Stirrup et al. (49) reported that the risk of ICU admission were equivalent. In addition, the differences in the risk of mortality mainly came from Funk et al. (31) and Martínez-García et al. (43) wherein they concluded that the mortality rate was reduced, and Stirrup et al. (49) concluded that the mortality rate was equivalent. For the Beta variant compared to wild-type virus, Funk et al. (31), Fisman and Tuite, (35), and Veneti et al. (46) found that it increased the risk of hospitalization and ICU admission. In addition, Funk et al. (31) and Fisman and Tuite, (35) reported that Beta variant also increased the risk of mortality. It is worth mentioning that Beta variant had the highest risk of hospitalization at 2.16 (95% CI: 1.19–3.14). The only study involved in the meta-analysis regarding the Delta variant was from Fisman and Tuite, (35). The risk of ICU admission and mortality were 3.35 (95% CI: 2.5–4.2) and 2.33 (95% CI: 1.45–3.21) respectively, and it was slightly lower than that of Beta variant in the risk of hospitalization at 2.08 (95% CI: 1.77–2.39)(35).

The results showed that in the risk of hospitalization, ICU admission, and mortality, all the SARS-CoV-2 VOCs had different degrees of increase compared with wild-type virus; Delta variant had the highest risk of ICU admission and mortality, and Beta variant had the highest risk of hospitalization.

## Discussion

Since the rapid spread of the SARS-COV-2 pandemic, many variant viruses including Alpha, Beta, Gamma, and Delta have emerged. However, the conclusions regarding disease severity of these variant viruses are not consistent. Accordingly, we searched for studies in the relevant field and recorded their clinical data. A meta-analysis was used to combine the information of different studies. Finally, we found that all VOCs increase the risk of hospitalization, ICU admission, and death compared with the wild-type virus, and variant Delta and Beta carried a much higher risk than other variants.

By comparing the results from different studies, we found that most of the conclusions stated that Alpha variant had a higher risk of disease severity than the wild-type virus, but Frampton et al. (25), Funk et al. (31), Martínez-García et al. (43), and Stirrup et al. (49) have expressed different opinions. However, the sample size of Frampton et al. (25) was very small, which is why their results were likely not very convincing. Although Frampton et al. (25) used whole genome sequencing to identify Alpha variant, while other reports used PCR detection of S-gene target failure (SGTF) as an alternative detection method, it did not make much of a difference to the results. Funk et al. (31) found that Alpha variant showed significantly higher risk of hospitalization rate and ICU admission, but lower risk of mortality than the wild-type. The clinical drugs for the Alpha variant in EU/EEA were more effective or some of the reported cases may have been vaccinated. The previously reported increased binding affinity between the spike receptor-binding domain and the angiotensin-converting enzyme 2 (ACE2) receptor in the Alpha SARS-CoV-2 strain may have led to further down-regulation of ACE2 if an individual got infected by this new variant compared with other variants. ACE2 was suggested to have a protective effect on lung injury in patients with COVID-19 (51, 52). Patients were aged ~70 years, and there was a large gap with the age of other studies' patients, which led to certain limitations regarding the conclusion of mortality risk (43). Stirrup et al. (49) concluded that female rather than male patients infected with the Alpha variant would have a higher risk of severe disease. In summary, it can be argued that Alpha variant was more threatening than the wild-type virus and can cause higher risk of more severe disease. The explanation for the conclusion from Funk et al. (31) that variants Beta and Gamma may also have a lower risk of mortality can be consistent with the explanation for Alpha variant. In addition, Hoang et al. (39) speculated about the risk of hospitalization and ICU admission by directly comparing variants Beta, Gamma, and Alpha. The risk of Beta variant was significantly higher than that of Alpha variant, while the risk of Alpha variant was similar to that of Gamma variant, which was consistent with our conclusion.

Although the sample size on the Delta variant was limited in conducting meta-analysis, those studies that directly compared the disease severity of variants Delta and Alpha supported our conclusions from the side. Using stratified Cox proportional hazard regression, there was a significantly increasing risk of hospitalization and emergency care attendance for Delta variant cases compared with Alpha variant cases after adjustment for confounders, which were 2.16 (95% CI: 1.56–4.36) and 1.67 (95% CI: 1.25–2.23), respectively (32). Ong et al. (42) calculated that the risk of ICU admission and mortality were 1.88 (95% CI: 0.95–3.76) and 1.88 (95% CI: 0.95–3.76), respectively. Among similar studies, the main debate involved the study from Frampton et al. (25), but as mentioned earlier, their work was limited by a much smaller sample size, which is why their conclusions were not very persuasive (21). The result of Kow et al. (22) in the risk of mortality for Alpha variant compared with the wild-type virus was 1.45 (95% CI: 1.18–1.78), which was close to our meta-analysis result at 1.37 (95% CI: 1.15–1.60). A recent review concluded that variants Alpha, Beta and Gamma all had a higher risk of hospitalization and ICU admission compared with the wild-type virus, and the risk of Beta variant was much higher (23), which supported our conclusion to some extent.

To our knowledge, this is the first study to compare the disease severity of VOCs with the wild-type virus and draw specific conclusions. We believe that our results present the threats of VOCs more clearly to the public, particularly the variants Beta and Delta. Although several different types of vaccines have been developed, further research is required regarding the protection rate of the viral variants. The fact that we did not further analyze the influence of age, sex, and geographic parameters is the limitation of the study. However, we performed meta-analysis, which is known to better reduce the impact of each study.

## Conclusion

In this meta-analysis, we analyzed the results of studies that reported on the disease severity of SARS-COV-2 VOCs from June 1, 2020 to October 15, 2021 and processed the relevant data. By comparing with the wild-type virus, in terms of the risk of hospitalization, ICU admission, and mortality, the variants Beta and Delta have a higher risk than the variants Alpha and Gamma, and all SARS-COV-2 VOCs have a higher risk of disease severity than the wild-type virus. This is the first comprehensive study that compared the disease severity of variants Alpha, Beta, Gamma and Delta with wild-type virus and drew specific conclusions. We hope that this report can increase the awareness of the disease severity of SARS-COV-2 VOCs, particularly of variants Beta and Delta, and make the public aware of routine precautions and the importance of vaccination.

## Data Availability Statement

The original contributions presented in the study are included in the article/supplementary material, further inquiries can be directed to the corresponding author/s.

## Author Contributions

All authors listed have made a substantial, direct, and intellectual contribution to the work and approved it for publication.

## Funding

This work described in this paper was partially supported by a grant from the Research Grants Council of the Hong Kong Special Administrative Region, China (HKU C7123-20G).

## Conflict of Interest

The authors declare that the research was conducted in the absence of any commercial or financial relationships that could be construed as a potential conflict of interest.

## Publisher's Note

All claims expressed in this article are solely those of the authors and do not necessarily represent those of their affiliated organizations, or those of the publisher, the editors and the reviewers. Any product that may be evaluated in this article, or claim that may be made by its manufacturer, is not guaranteed or endorsed by the publisher.
